# Expanding diversity of bunyaviruses identified in mosquitoes

**DOI:** 10.1038/s41598-023-45443-2

**Published:** 2023-10-24

**Authors:** Yasuko Orba, Yusuf Eshimutu Abu, Herman M. Chambaro, Tapiwa Lundu, Walter Muleya, Yuki Eshita, Yongjin Qiu, Hayato Harima, Masahiro Kajihara, Akina Mori-Kajihara, Keita Matsuno, Michihito Sasaki, William W. Hall, Bernard M. Hang’ombe, Hirofumi Sawa

**Affiliations:** 1 Division of Molecular Pathobiology, International Institute for Zoonosis Control, Hokkaido University, N20, W10, Kita-ku, Sapporo, 001-0020 Japan; 2 International Collaboration Unit, International Institute for Zoonosis Control, Hokkaido University, Sapporo, Japan; 3 Institute for Vaccine Research and Development, Hokkaido University, Sapporo, Japan; 4 One Health Research Center, Hokkaido University, Sapporo, Japan; 5 Department of Biomedical Sciences, School of Veterinary Medicine, University of Zambia, P.O. Box 32379, 10101 Lusaka, Zambia; 6 Virology Unit, Central Veterinary Research Institute, Lusaka, Zambia; 7 Division of Collaboration and Education, International Institute for Zoonosis Control, Hokkaido University, Sapporo, Japan; 8 Division of International Research Promotion, International Institute for Zoonosis Control, Hokkaido University, Sapporo, Japan; 9 Division of Global Epidemiology, International Institute for Zoonosis Control, Hokkaido University, Sapporo, Japan; 10 Division of Risk Analysis and Management, International Institute for Zoonosis Control, Hokkaido University, Sapporo, Japan; 11 National Virus Reference Laboratory, University College Dublin, Belfield, Dublin, 4, Ireland; 12 Global Virus Network, Baltimore, MD USA; 13 Department of Para-Clinical Studies, School of Veterinary Medicine, University of Zambia, Lusaka, Zambia; 14 Africa Centre of Excellence for Infectious Diseases of Humans and Animals, Lusaka, Zambia

**Keywords:** Biological techniques, Microbiology, Environmental sciences

## Abstract

Mosquitoes interact with various organisms in the environment, and female mosquitoes in particular serve as vectors that directly transmit a number of microorganisms to humans and animals by blood-sucking. Comprehensive analysis of mosquito-borne viruses has led to the understanding of the existence of diverse viral species and to the identification of zoonotic arboviruses responsible for significant outbreaks and epidemics. In the present study on mosquito-borne bunyaviruses we employed a broad-spectrum RT-PCR approach and identified eighteen different additional species in the *Phenuiviridae* family and also a number of related but unclassified bunyaviruses in mosquitoes collected in Zambia. The entire RNA genome segments of the newly identified viruses were further analyzed by RNA sequencing with a ribonuclease R (RNase R) treatment to reduce host-derived RNAs and enrich viral RNAs, taking advantage of the dsRNA panhandle structure of the bunyavirus genome. All three or four genome segments were identified in eight bunyavirus species. Furthermore, L segments of three different novel viruses related to the *Leishbunyaviridae* were found in mosquitoes together with genes from the suspected host, the *Crithidia* parasite. In summary, our virus detection approach using a combination of broad-spectrum RT-PCR and RNA sequencing analysis with a simple virus enrichment method allowed the discovery of novel bunyaviruses. The diversity of bunyaviruses is still expanding and studies on this will allow a better understanding of the ecology of hematophagous mosquitoes.

## Introduction

Bunyaviruses are segmented negative-strand RNA viruses. As of 2022, based on the current International Committee on Taxonomy of Viruses (ICTV) taxonomy release, the order *Bunyavirales* harbors 14 families, 4 subfamilies, and 63 genera. The largest family *Phenuiviridae* consists of 22 genera, including the clinically important *Phlebovirus* and *Bandavirus*, a total of 151 species as well as many unclassified viruses. Recent comprehensive genome analyses have accelerated the discovery of phenuiviruses from highly diverse organisms, such as vertebrate and invertebrate animals, plants, protozoa, and fungi^1–4^.

Viruses that exist in hematophagous arthropods, such as mosquitoes, sandflies, and ticks, are particularly noteworthy as these vectors can potentially transmit arboviruses to mammals. One of the mosquito-borne arboviruses, *Phlebovirus riftense* (Rift Valley fever virus) causes a severe illness, Rift Valley fever (RVF) in ruminants and humans in Africa^5^. Zambia had already experienced RVF epizootics more than three decades ago^6–8^. Although several environmental factors are thought to have brought about an unusually long inter-epizootic/-epidemic period in Zambia, silent circulation of RVFV in wild and domestic ruminants and the risk of disease emergence certainly still occurs in some areas^9^. In the present study, surveillance methods employing a broad-spectrum RT-PCR targeting the bunyavirus RNA-dependent RNA polymerase (RdRP) genes and RNA sequencing analysis was conducted to better understand the extent of arboviruses infections, including RVFV, and to simultaneously uncover unknown viruses in mosquitoes.

Metagenomic analysis of host or environmental samples allows the discovery of known and unknown microorganisms. To identify low–titer viruses in a complex mix of predominantly host genes/transcripts employing high throughput sequencing, viral particle/nucleic acid enrichment approaches, such as filtration, nuclease digestion prior to total nucleic acid extraction, and negative or positive selection-based enrichment of viral genes, are required for sample preparation^10^. However, for non-model organisms, removal of host RNAs remains challenging. Ribonuclease R (RNase R), which is a 3' to 5' exonuclease that digests linear RNAs^11^, has recently been used to detect circular RNAs derived from cellular transcripts or viral genes^12–14^. We applied RNase R treatment to reduce most linear cellular RNAs and enrich viral genomes containing double-stranded (ds) RNA structures at their 3'-ends. Bunyavirus genomes with a dsRNA panhandle structures at the 3'- and 5'-ends would be left undigested and enriched from total mosquito RNA by RNase R treatment. This virus detection approach employing broad-spectrum RT-PCR followed by the total RNA sequencing with a simple and inexpensive virus gene enrichment step has now efficiently allowed the discovery of new and diverse bunyavirus genes in mosquitoes.

## Results

### Detection of diverse bunyaviruses within the *Phenuiviridae* and unclassified bunyaviruses

Field mosquitoes were collected from various locations in Zambia from 2014 to 2022. A total of 17,281 adult female mosquitoes were divided into 964 pools according to species for the purpose of detection and isolation of bunyaviruses (Supplemental Table S1). The broad-spectrum RT-PCR assay designed to detect a wide range of arbovirus species within *Phenuiviridae* was applied to RNA extracted from the mosquitoes and this allowed the detection of bunyavirus sequences from 150 pools^9^. BLAST analyses of sequences of the PCR products identified various bunyavirus-like sequences with some similarity to previously-identified viral RNA-dependent RNA polymerase (RdRP) genes (Table 1).

**Table 1:** Bunyavirus-positive mosquito species collected in Zambia.

Year	Month	Place	Species	No. mosquito	No. pools	RT-PCR (+) pools	Closest virus by BLAST (nt or aa Identity %)	Pool# and virus registered in this study
2014	Apr	Lusaka	*Culex quinquefasciatus*	106	5	1	#33 Culex Bunyavirus 2 (nt 87%)	
2014	Jul	Lusaka	*Culex quinquefasciatus*	257	9	1	#4 Culex Bunyavirus 2 (nt 87%)	
2014	Oct	Mongu	*Culex quinquefasciatus*	1301	43	3	#13,45 Culex Bunyavirus 2 (nt 75%) #58 Cumuto virus (nt 55%)	#45 Culex bunyavirus 2 #58 Culex goukovirus 2
2015	Feb	Lusaka	*Culex quinquefasciatus*	440	15	3	#1,14,13 Culex Bunyavirus 2 (nt 87%)	
2015	Mar	Lusaka	*Culex quinquefasciatus*	1050	32	4	#15,29,33,34 Culex Bunyavirus 2 (nt 87%)	
2015	June	Lusaka	*Culex quinquefasciatus*	70	3	1	#3 Culex Bunyavirus 2 (nt 87%)	#3 Culex bunyavirus 2
2015	Apr	Livingstone	*Culex quinquefasciatus*	773	26	26	Culex Bunyavirus 2 (nt 87%) *	#15 Culex bunyavirus 2 #15 Culex leishbunyavirus 1 ** #15 Culex leishbunyavirus 2 **
			*Anopheles funestus*	2	1	1	#16 Badu virus (nt 70%) *	#16 Anopheles phasivirus 2
2015	Apr	Kazungula	*Culex quinquefasciatus*	45	5	1	#36 Culex Bunyavirus 2 (nt 87%)	
			*Anopheles funestus*	13	2	2	#29,33 Xinzou bunya-like virus (nt 76%) *	#29 Anopheles phasivirus 2 ** #29 Anopheles bunyavirus 1
2016	Mar	Siavonga	*Anopheles rufipes*	2	2	1	#40 Badu virus (aa 67%) *	#40 Anopheles phasivirus 1
2016	May	Mongu	*Culex quinquefasciatus*	278	15	1	#15 Culex Bunyavirus 2 (nt 75%)	#15 Culex bunyavirus 2
			*Culex univittatus*	126	5	1	#55 Wenling crustacean virus 7 (aa 40%)	#55 Culex phenuivirus 2
			*Anopheles coustani*	182	9	1	#25 Xinzou mosquito virus (nt 78%)	#25 Anopheles bunyavirus 1
			*Coquillettidia fuscopennata*	121	7	2	#18,19 Xinzou bunya-like virus 1 (aa 36%)	#18 Coquillettidia phenuivirus
2016	Nov	Mwinilunga	*Culex quinquefasciatus*	36	4	2	#21 Gouleako virus (nt 77%) * #23 Culex Bunyavirus 2 (nt 87%)	#21 Culex goukovirus 1 #21 Herbevirus herberti**
2017	Apr	Livingstone	*Culex quinquefasciatus*	780	34	29	Culex Bunyavirus 2 (nt 87%)	#1 Culex bunyavirus 2
			*Anopheles funestus*	16	1	1	#46 Xinzhou mosquito virus (nt 75%)	#46 Anopheles bunyavirus 1
			*Anopheles squamosus*	34	1	1	#51 Bunya enviromental (nt 68%)	#51 Anopheles bunyavirus 2
			*Anopheles* sp.	1	1	1	#74 Badu virus (nt 68%)	#74 Anopheles phasivirus 2
			*Aedes* sp.	7	3	1	#4 Bunya enviromental (nt 68%)	#4 Aedes bunyavirus
2017	May	Mongu	*Culex univittatus*	309	15	2	#17 Wenling crustacean virus 7 (aa 50%) * #117 Wenling crustacean virus 7 (aa 39%)	#17 Culex phenuivirus 1 #117 Culex phenuivirus 2
			*Coquillettidia fuscopennata*	46	5	1	#56 Xinzhou bunya-like virus 1 (aa 36%)	#56 Coquillettidia phenuivirus
			*Anopheles coustani*	709	29	5	#43,68,69,70,134 Xinzhou Mosquito Virus (aa 92%)	#134 Anopheles bunyavirus 1
			*Anopheles squamosus*	173	8	2	#58,104 Phasi like virus (nt 68%)	#104 Anopheles phasivirus 1
			*Anopheles* sp.	555	24	1	#74 Badu virus (nt 70%)	#74 Anopheles phasivirus 2
2017	Nov	Isoka	*Culex quinquefasciatus*	97	9	7	Culex Bunyavirus 2 (nt 87%)	
			*Coquillettidia fuscopennata*	68	6	2	#22 Bunya enviromental (nt 64%) #41 Badu virus (nt 82%)	#22 Coquillettidia bunyavirus
			*Coquillettidia* sp.	15	2	1	#43 Xinzhou bunya-like virus 1 (aa 36%)	#43 Coquillettidia phenuivirus
2017	Nov	Mpulungu	*Culex quinquefasciatus*	359	20	2	Culex Bunyavirus 2 (nt 87%)	
			*Aedes aegypti*	8	5	1	#71 Phasi like virus (aa 46%)	#71 Aedes phasivirus
2018	Dec	Livingstone	*Culex quinquefasciatus*	587	25	24	Culex Bunyavirus 2 (nt 87%)	
2018	Dec	Mongu	*Culex quinquefasciatus*	787	31	1	#50 Hubei lepidoptera 1 (aa 49%)*	#50 Culex hudovirus #50 Culex pheuivirus 3 ** #50 Culex leishbunyavirus 3 ** #50 Kristianstad virus **
			*Mansonia uniformis*	392	16	1	#48 Hubei lepidoptera 1 (aa 49%)	
2019	May	Mongu	*Culex quinquefasciatus*	824	30	6	#45,46,50,54,60 Rice grassy stunt virus (nt 69%) * #51 Yichang insect virus (nt 70%)	#45,46,50,54,60 Culex tenui-like virus #51 Culex goukovirus 3
			*Anopheles coustani*	300	13	4	#26,28,73,75 Cx bunya-like virus (nt 79%)	#73,75 Anopheles bunyavirus 1
			*Mansonia* sp.	174	9	2	#34 Xinzhou Mosquito virus (nt 70%) #35 Gouleako virus (nt 71%)	#34 Mansonia bunyavirus #35 Mansonia goukovirus
			*Coquillettidia aurites*	5	3	1	#40 Bunya environmental (nt 73%)*	#40 Coquillettidia bunyavirus
			*Coquillettidia fuscopennata*	60	4	2	#84 Bunya environmental (nt 72%) #65 Wenlling crustacean virus 7 (aa 41%)	#65 Coquillettidia phenuivirus
2022	Apr	Lusaka	*Culex quinquefasciatus*	449	14	1	Culex Bunyavirus 2 (nt 87%)	

*Total RNA sequencing was performed.

**Identified only by RNA sequencing analyses.

**Figure 1:**
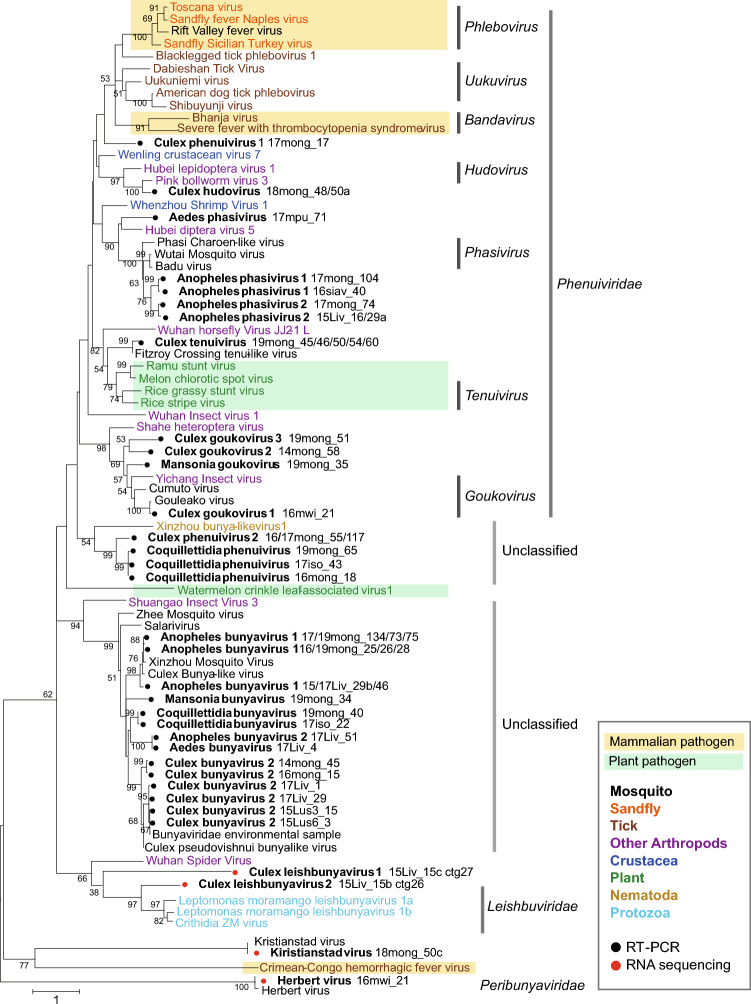
Molecular phylogenetic analysis of partial RdRP sequences. The maximum likelihood-based phylogenetic tree was constructed using partial RdRP amino acid sequences of representative bunyaviruses and identified genes in this study. Bootstrap values higher than 50 are shown adjacent to the tree branches. The tree is drawn to scale with branch lengths representing the number of substitutions per site. Black circles indicate bunyaviruses identified by the broad-spectrum RT-PCR. Bunyavirus contigs identified from RNA sequencing analyses are marked with red circles. The organisms in which the viruses were identified are color-coded with a caption in the box at the right lower corner of the Figures. The viruses considered to be mammalian pathogens or plant pathogens are highlighted in light orange and light green colors, respectively.

The phylogenetic analysis of partial RdRP amino acid sequences showed that ten different viral sequences were identified in the *Phenuiviridae* clade; tentatively named as Culex (Cx) phenuivirus 1, Cx hudovirus, Cx tenuivirus, Cx goukovirus 1, 2, and 3, Aedes (Ae) phasivirus, Anopheles (An) phasivirus 1 and 2, and Mansonia goukovirus (Fig. 1). Two new species, Cx phenuivirus 2 and Coquillettidia (Cq) phenuivirus formed a cluster with Xinzhou bunya-like virus 1 (Accession No. KX884868), and which had a 36% amino acid identity with the Cq phenuivirus. The other six species, Cx bunyavirus 2, Ae bunyavirus, An bunyavirus 1 and 2, Cq bunyavirus, Mansonia bunyavirus were found to cluster with previously identified unclassified bunyaviruses **(**Fig. 1**)**.

### Regional difference of Cx bunyavirus 2 prevalence in* Culex quinquefasciatus* in Zambia

**Figure 2:**
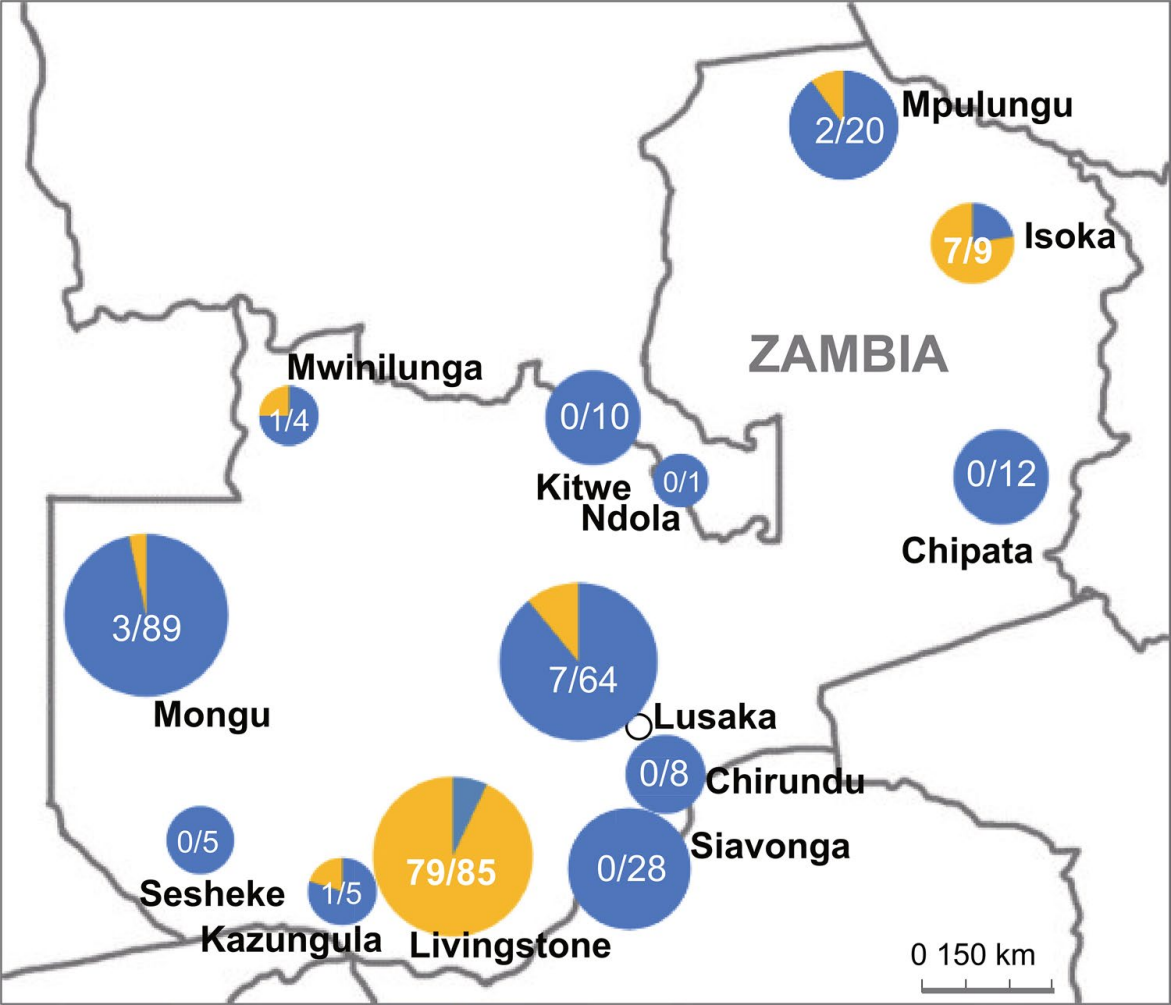
Prevalence of the Culex bunyavirus 2 in the *Culex quinquefasciatus* at each district in Zambia. The pie charts indicate the number of Culex bunyavirus 2-positive pools (yellow)/the pool number of *Culex quinquefasciatus* collected in each district shown on the map of Zambia.

Among the 150 bunyavirus-positive pools, sequences of the PCR products from 103 *Culex quinquefasciatus* (*Cx. quinquefasciatus*) pools were closely related to Cx bunyavirus 2 (Accession No. MH188052) in the unclassified bunyavirus group with 87% nucleotide identity in the partial RdRP gene. As these new strains of Cx bunyavirus 2 were highly prevalent in *Cx. quinquefasciatus*, we further analyzed regional differences of the prevalence of Cx bunyavirus 2 infection (Table 2 and Fig. 2). The Cx bunyavirus 2 positivity was especially high in *Cx. quinquefasciatus* present in Livingstone (79/85 pools, minimum infection rate per 1000: 33.64–40.89) and Isoka (7/9 pools, minimum infection rate per 1000: 75.27) districts compared to other regions. The *Cx. quinquefasciatus* mosquito is the most common mosquito species in Zambia and indeed high numbers of *Cx. quinquefasciatus* were collected in all regions of the country. Although environmental factors such as rainfall, temperature, and animal populations that are characteristic of Livingston or Isoka have not been identified^9^, the obtained results suggest that regionally different factors may be involved in the transmission of Cx bunyavirus 2.

**Table 2:** Prevalence of the Culex bunyavirus 2 in the *Culex quinquefasciatus* in Zambia.

Year	Month	Place	No. mosquitoes	No. pools	No. Cx bunyavirus positive pools (%)	Minimum infection rate per 1000*	CI (95%)**
2014	Apr	Lusaka	106	5	1 (20.0%)	9.43	0.00–27.84
2014	Jun–Jul	Lusaka	257	9	1 (11.1%)	3.89	0.00–11.50
2015	Feb	Lusaka	440	15	3 (20.0%)	6.82	0.00–14.51
2015	Mar	Lusaka	1050	32	4 (12.5%)	3.81	0.08–7.54
2015	June	Lusaka	70	3	1 (33.3%)	14.29	0.00–42.08
2015	Apr	Livingstone	773	26	26 (100.0%)	33.64	20.93–46.34
2017	Apr	Livingstone	780	34	29 (85.3%)	37.18	23.90–50.46
2018	Dec	Livingstone	587	25	24 (96.0%)	40.89	24.86–56.91
2015	Apr	Kazungula	45	5	1 (20.0%)	22.22	0.00–65.29
2016	Mar	Chirundu	160	8	0	0.00	
2014	Apr	Siavonga	532	18	0	0.00	
2016	Mar	Siavonga	224	10	0	0.00	
2018	Dec	Sesheke	79	5	0	0.00	
2014	Oct	Mongu	1301	43	2 (4.7%)	1.54	0.00–3.67
2016	May	Mongu	278	15	1 (6.7%)	3.60	0.00–10.63
2017	May	Mongu	285	22	0	0.00	
2018	Dec	Mongu	787	31	0	0.00	
2016	Nov	Kitwe	275	10	0	0.00	
2016	Nov	Ndola	15	1	0	0.00	
2016	Nov	Mwinilunga	36	4	1 (25.0%)	27.78	0.00–81.46
2015	Nov	Chipata	286	12	0	0.00	
2017	Nov	Isoka	97	9	7 (77.8%)	75.27	21.64–128.88
2017	Nov	Mpulungu	359	20	2	5.57	0.00–13.27
		Total	8818	362	103 (28.5%)		

*The infection rate in the *Culex quinuquefasciatus* was calculated by the maximum likelihood estimation.

**The confidence interval (CI, 95%) was indicated in the range between the upper and lower bounds.

### Viral RNA enrichment for mosquito RNA sequencing by RNase R treatment

**Figure 3:**
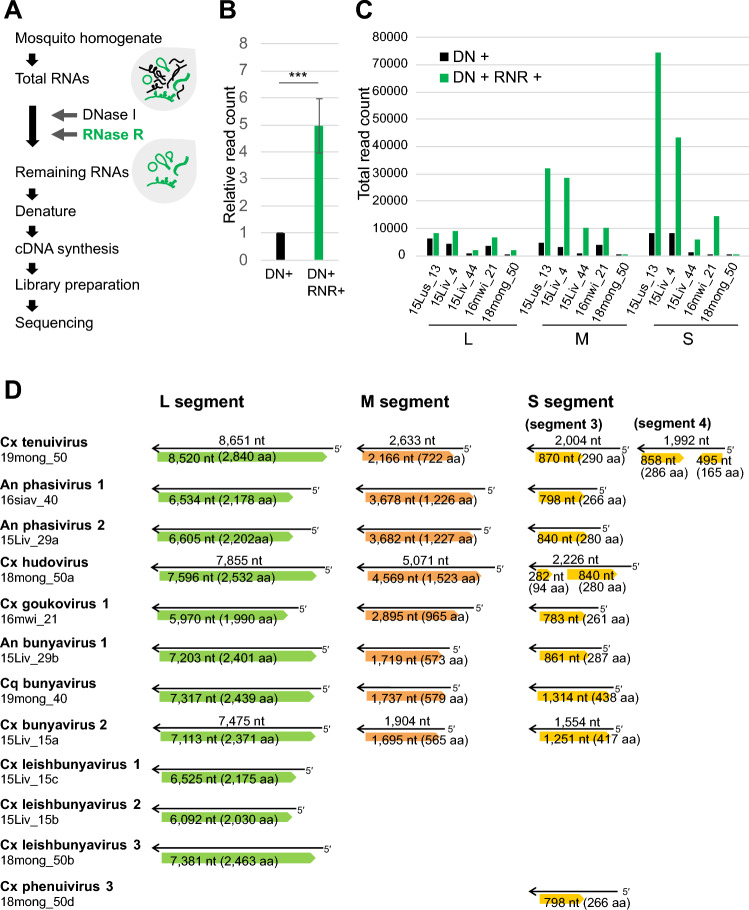
Identification of viral genomes by total RNA sequencing of mosquito RNAs with RNase R treatment. (**A**) Library preparation for bunyavirus RNA enrichment. (**B**) Relative read counts of bunyavirus RNAs on contigs > 1000 bases. *DN+* DNase I treatment, *RNR+* RNase R treatment. Data represents the mean (± SD) of five samples. *** *t*-test, two-sided test, *p* = 0.0009. (**C**) Total reads on bunyavirus tripartite segments in each sample. (**D**) Schematic representation of the genome segments and predicted coding regions are shown for bunyaviruses identified from total RNA sequencing. Asterisk: partial sequence.

Virus isolation assays of these viruses were attempted using several mosquito and mammalian cell lines, however, no productive viral growth was observed. To identify the entire tripartite RNA genomes of the detected bunyaviruses, we performed RNA sequencing analysis of the total RNA from the virus-positive mosquito lysates with a viral RNA enrichment step (Fig. 3A). In addition to the digestion of DNAs with DNase I, total RNA was treated with RNase R, which is a 3' to 5' exoribonuclease that digests linear RNAs, including cellular mRNAs, but does not digest lariat or circular RNA structures or double-stranded (ds) RNA with 3' overhangs shorter than seven nucleotides. Therefore, the bunyavirus genome, in which the 3' and 5' ends of each genome segment are highly complementary and form a dsRNA stem structure^15^, are expected to be resistant to RNase R treatment. First, we compared the number of bunyavirus reads from five representative RNA samples treated with or without RNase R. Although three segments of bunyavirus contigs longer than 1000 bases were obtained from both DNase I alone and DNase I and RNase R-treated samples, the number of reads on the bunyavirus contigs increased in the samples treated with RNase R compared to that with DNase I alone (Fig. 3B, C). These results indicate that RNase R treatment can enrich bunyavirus genes from total RNAs consisting of mostly host mosquito RNAs and this increases the reads of viral gene sequences (Supplementary Fig. S1).

### Identification of bunyavirus genome segments with conserved protein coding sequences

**Figure 4:**
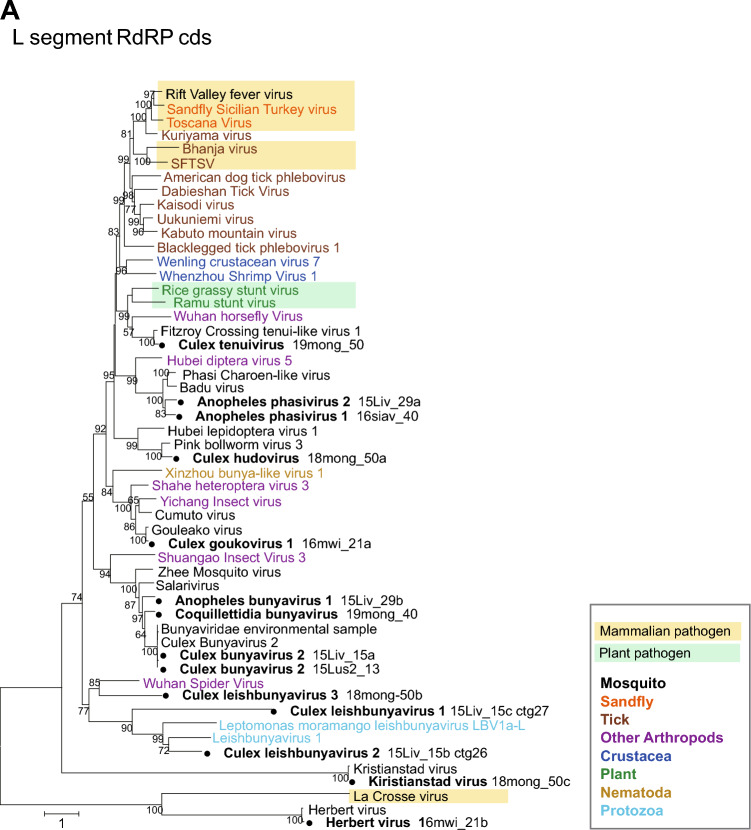

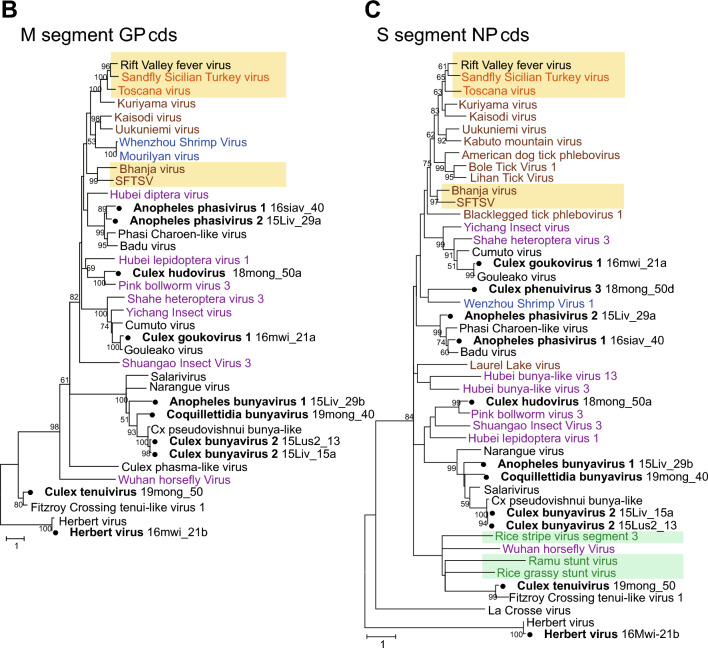
Molecular phylogenetic analysis of the coding sequence of viral proteins. Maximum likelihood-based phylogenetic trees were generated from (**A**) the RdRP coding sequences on the L segment, (**B**) Glycoprotein (GP) coding sequences on the M segment, and (**C**) nucleoprotein (NP) sequences on the S segment. Bootstrap values higher than 50 are shown adjacent to the tree branches. The tree is drawn to scale with branch lengths representing the number of substitutions per site. Viral sequences identified from mosquitoes in this study are marked with black circles. The organisms in which the viruses were identified are color-coded with a caption in the box at the right lower corner of the Figures. The viruses considered to be mammalian pathogens or plant pathogens are highlighted in light orange and light green colors, respectively.

The RNA sequencing and BLASTx analyses eventually identified all three or four segments of the virus genome in eight bunyavirus species. As a result of attempting to identify the terminal sequences of these bunyaviruses by RACE analysis, we were able to determine the complete genome sequences only for Cx tenuivirus, Cx hudovirus and Cx bunyavirus 2 (Fig. 3D). The phylogenetic analyses of the putative coding sequences of RdRP on the L segment, glycoprotein on the M segment, and nucleoprotein sequences on the S segment were performed (Fig. 4A–C). RdRP and nucleoprotein sequences of Cx tenuivirus clustered with sequences of the genus *tenuivirus,* which has four segments of viral genome and infects host plants and vector insects. The putative glycoprotein on the M segment was found in Cx tenuivirus as well as previously identified Fitzroy Crossing tenui-like virus 1^16^, although glycoproteins of previously detected tenuiviruses have not been identified. The phylogenetic analysis of bunyavirus glycoproteins showed that Culex tenuivirus and Fitzroy Crossing tenui-like virus 1 have unique glycoprotein-like proteins distinct from the other bunyaviruses (Fig. 4B). In some samples, only the L and/or S segment of bunyavirus were identified by BLAST analysis. Three different L segments of bunyaviruses related to the *Leishbunyaviridae* (Cx leishbunyavirus 1, 2, and 3) and a putative S segment (Cx phenuivirus 3) were discovered in the Culex mosquito RNA samples. As bunyaviruses in the *Leishbunyavirudae* have been identified from protozoa in the family *Trypanosomatidae*^17^, we further explored protozoa genes in the RNA sequencing data in which Cx leishbunyavirus genes were found. As expected, contigs of *Crithidia fasciculata* ribosomal DNA gene were detected in the pool of *Cx. quinquefasciatus* (2015_Livingston_#15, Table 1) that contains two species of leishbunyaviruses, suggesting that these leishbunyaviruses may be derived from *Crithidia fasciculata* (Supplementary Table S2 and S3)*.* Additionally, in the *bunyavirales,* RNA sequencing analyses in this study identified the L segment of previously known unclassified bunyaviruses, Kiristianstad virus from a pool of *Cx. quinquefasciatus* (18mong_50), and all three segments of Herbert virus in the *Peribunyaviridae* from a pool of *Cx. quinquefasciatus* (16mwi_21) **(**Fig. 4A–C**)**.

## Discussion

This study has revealed the existence of diverse bunyaviruses which have adapted to each mosquito species. The broad-spectrum RT-PCR assay designed to target the highly conserved region of RdRP gene within phleboviruses, including RVFV, allowed the detection of a wide variety of *phenuiviridae*-related viruses, including those plant-associated viruses. Thus, the broad-spectrum RT-PCR targeting the conserved RdRP gene is convenient for comprehensive detection of known arboviruses and unknown viruses in vector mosquitoes, ticks^18^, and other biological samples.

The transmission cycle of the identified viruses is still unknown. Further investigation of animals and environmental samples and isolation of the viruses are required to clarify the individual viral life cycles, whether mosquitoes are a reservoir or just a transient vector, and the zoonotic potential of newly identified bunyaviruses. One of the discovered viruses, Cx tenuivirus with characteristic four genome segments, is closely related to the Genus *tenuivirus,* suggesting a possible plant host. When the adult mosquitoes feed on plant nectars, viruses can be transmitted horizontally between mosquitoes and plants. Interestingly, we found that Cx bunyavirus 2 was highly prevalent in *Cx. quinquefasciatus* which inhabit Livingstone and Isoka districts compared to other regions in Zambia. It is possible that the presence of other hosts of Cx bunyavirus 2, which is involved in the life cycle of *Cx. quinquefasciatus*, may be associated with the regional differences in virus positivity. The Livingstone City has a tourist spot of The Victoria Falls World Heritage Site/ Mosi-oa-Tunya National Park. A notable mosquito ecology in Livingstone which is the main source of *Cx. quinquefasciatus* appears to be a large waste stabilization pond covered with exotic water hyacinths adjacent to the National Park. It is likely that Livingstone has an ecological niche that contain non-mosquito hosts for Cx bunyavirus 2, and possibly other aquatic organisms in the aquatic stage of mosquito larvae.

In total RNA sequencing analysis, it is often difficult to detect very small amounts of viral genes in host total RNAs. The poly-A selection method is not applicable for enrichment of viral RNAs except for viruses with a poly-A tailed genome. Commercially available ribosomal RNA (rRNA) depletion systems target human, mouse, rat, and bacteria, and therefore species-specific probe preparations are required to deplete the rRNA from the total RNA of non-model animals. The results of RNA sequencing showed that our RNase R treatment system is very useful for the enrichment of bunyavirus genomes which have a dsRNA panhandle structure at the 3'- and 5'-ends of each genome segment, although it is difficult to rule out the possibility that the RNase R resistance may involve not only the double-stranded structures but also viral RNA protection by viral nucleoproteins or binding of the L protein to the 3' and 5' termini^19,20^. In addition, we found that the RNase R treatment system enriched not only bunyaviruses but also flaviviruses, which are positive sense ssRNA virus with higher order structures at their RNA 3'-ends^21^, and dsRNA viruses such as reoviruses. However, reads of ssRNA viruses with poly-A tail in their genome, such as *ifraviridae,* were decreased by RNase R treatment as expected (Supplementary Fig. S1). These results indicated that viral RNA genomes which have double-stranded or higher order structures at their RNA 3'-ends are enriched by RNase R digestion. Thus, the RNase R treatment approach has the advantage for the detection of RNA viruses, such as bunyavirus, flavivirus, and reovirus family members, in a variety of samples. It is expected to further improve the efficiency of virus detection by combinations of this simple and low-cost method with other virus enrichment methods and virome analysis pipelines to efficiently detect unknown viral genomes^10,22–24^. For some bunyaviruses, identification of all genome segments was unsuccessful. Besides a low copy number of viral genome in mosquitoes, it is possible that the viral genome could not be recognized by BLAST analysis due to low homology with known viral genes whose sequences have been registered. In particular, the M and S segments are less conserved than the RdRP-coding L segment, making them difficult to identify. For instance, the predicted nucleoprotein sequence (266 aa) of the Cx phenuiviurs 3, whose L and M segment were not found, have only 35% amino acid identity with previously known bunyaviruses. Although tick-borne phleboviruses without a M segment have been reported^25^, virus isolation is necessary to identify the characteristics of such novel bunyaviruses. The M and S segments of Cx leishbunyavirus 1, 2, and 3 were also missing, while previously reported leishbunyaviruses from *Crithidia* spp. have putative M and S segments^17^. In order to elucidate the relationship between these leishbunya-like viruses, *Crithidia* parasite, and *Culex* mosquitoes, and their role or pathogenicity, isolation of virus together with *Crithida* parasite will be absolutely necessary. Some phenuiviruses have an ambisense coding strategy and encode nucleoproteins and nonstructural proteins (NSs) on the S segment. Short open reading frames (approximately 100 aa) in the antigenomic direction of the S segment genes were also found in some of the identified bunyaviruses. However, amino acids sequences of these open reading frames have no homology with the NSs of phenuiviruses, and BLAST analysis failed to find any similar or related proteins.

The newly discovered viruses identified in this study indicate that field-collected mosquitoes harbor highly divergent bunyaviruses. Further investigation of other hosts and study of the ecological niches occupied by these novel bunyaviruses will improve our understanding of the viral evolution and their pathogenic and zoonotic potentials.

## Methods

### Mosquito collection

Mosquito collections were conducted between 2014 and 2022 with permission from the Department of National Parks and Wildlife, Ministry of Tourism and Arts of the Republic of Zambia, Excellence in Research Ethics and Science (ERES) converge ethics committee (IRB No: 00005948), and University of Zambia Biomedical research ethics committee (REF. NO. 1382-2020)^26^. We established three to five CDC light traps (John W. Hock Co., USA) with yeast fermentation to supply CO_2_, and three BG-sentinel traps (Biogents AG, Germany) at different locations in inhabited area. The traps were set in the afternoon and left until the following morning, over a period of five nights on average in each district. After species identification, one to up to 40 female mosquitoes were pooled from each species and stored at −80 °C. Mosquitoes were first identified morphologically with reference to the African mosquitoes identification keys^27–29^. For mosquitoes that could not be identified morphologically, DNA extracted from the mosquitoes was genetically confirmed via sequencing of the cytochrome oxidase I (COI) gene^30^ of mosquito DNA as a standard DNA barcoding for molecular identification.

### Screening of phenuivirus genes by broad-spectrum RT-PCR

Pooled mosquitoes were homogenized in Minimum Essential Medium containing 2% fetal bovine serum using the BioMasher (Nippi, Japan). RNA was extracted from 100 µL of supernatants of mosquito homogenates using the Direct-Zol kit (Zymo research, USA) according to the manufacturer’s instructions. The remaining mosquito homogenates were filtered and inoculated onto cells for virus isolation. To detect multiple phleboviruses, we designed degenerate primer sets targeting the conserved RdRP gene region; L-2779F 5'-CARCATGGWGGTYTDAGRGARATCTA-3' and L-3287R 5'-TGCARKATKCCYTGCATCATHCCWG-3′^9^. RNA samples were amplified using a PrimeScript One-step RT-PCR kit Ver.2 (Takara, Japan) and 1 µM of primer sets. The cycling protocol was comprised of 30 min of incubation at 50 °C for cDNA synthesis, followed by 2 min of incubation at 94 °C, 43 cycles each of 94 °C for 30 s, 52 °C for 30 s and 72 °C for 30 s, and 72 °C for 5 min. The PCR products were sequenced using a BigDye Terminator v3.0 Cycle Sequencing kit on an ABI PRISM 3130 Genetic Analyzer (Applied Biosystems, USA).

### Molecular phylogenetic analysis

Deduced amino acid sequences of pan-phlebovirus RT-PCR products, or coding regions of the L, M or S segments were aligned with previously-characterized bunyaviruses (Supplementary Table S4) using the ClustalW^31^. Maximum likelihood-based phylogenetic tree search was performed based on the Le_Gascuel_2008 model using MEGA7 with 1000 bootstrap replicates^32,33^.

### Viral RNA sequencing

Viral genome sequences of bunyaviruses were identified by Illumina dye sequencing. The total RNA extracted from mosquito homogenates was treated with or without 30 U of RNase R (Epicenter Biotechnologies, USA) for 15 min at 37 °C. Reaction solutions were then digested with DNase I in the RNA Clean & Concentrator column (Zymo research). Double-stranded cDNAs were transcribed from the RNase R and DNase I-treated RNAs using the PrimeScript ds-cDNA Synthesis kit (Takara). One nanogram of the ds-cDNA was used for library preparation using the Nextera XT DNA Library Prep (Illumina, USA), followed by sequencing with a MiSeq Reagent Kit v3 (600 cycles) and an Illumina MiSeq System. De novo assembly of virus genomes was achieved using the CLC Genomics Workbench 10 (CLC bio, Qiagen, Germany). Virus-associated contigs longer than 500 or 1,000 nucleotides were identified by BLASTn and BLASTx searches against viral genes in the National Center for Biotechnology Information (NCBI) database.

RACE analyses of the 5'- and 3'-sequences at the ends of the bunyavirus RNA genomes were performed using a 5'-Full RACE Core Set (Takara) or SMARTer RACE 5'/3' kit (Takara) according to the manufacturer’s instructions. A poly-A tail was ligated to the isolated RNA using E. coli poly (A) polymerase prior to cDNA synthesis in order to amplify the 3'-ends using the SMARTer RACE system. Identified terminal sequences were confirmed by Sanger sequencing.

### Minimum infection rate analysis

The minimum infection rate (MIR) was estimated as the number of infected mosquitoes per 1,000 mosquitoes, with the bias corrected by the maximum likelihood estimator, with a confidence interval of, 95%, using the program PooledInfRate v.4.0^34^.

### Supplementary Information

Supplementary Information.

## Data Availability

All sequences generated in the present study were deposited in the DNA Data Bank of Japan (DDBJ) under the accession numbers and BioSamples described in Supplemental Table S4, and BioProject (PRJDB16048).
