# Supplementary material for: Expanding diversity of bunyaviruses identified in mosquitoes
Source: Sci Rep. 2023 Oct 24;13:18165. doi: 10.1038/s41598-023-45443-2 (PMC10598057; doi:10.1038/s41598-023-45443-2)
Supplement: Supplementary file 1 — Supplementary Information. [file 41598_2023_45443_MOESM1_ESM.pdf]

Supplementary Information

Expanding diversity of bunyaviruses identified in mosquitoes

Yasuko Orba, Yusuf Eshimutu Abu, Herman M. Chambaro, Tapiwa Lundu, Walter Muleya, Yuki Eshita, Yongjin Qiu, Hayato Harima, Masahiro Kajihara, Akina Mori-Kajihara, Keita Matsuno, Michihito Sasaki, William W. Hall, Bernard M. Hang'ombe, Hirofumi Sawa.

Supplementary Figure S1

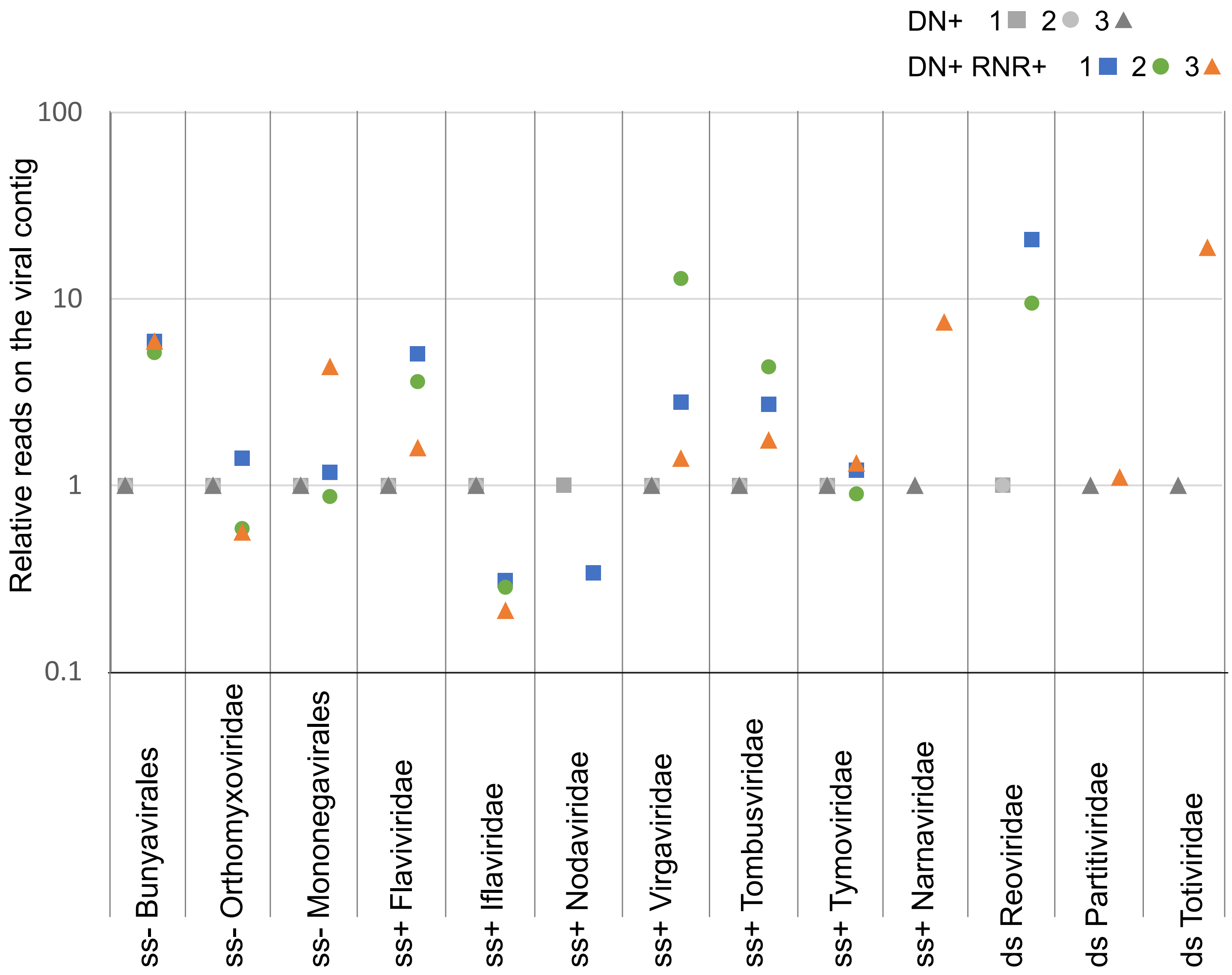

Supplementary Figure S1.
Relative read counts on the each viral contig was analyzed in the RNA sequencing data of three representative RNA samples (1: 15Liv_44, 2: 15Liv_4, 3: 15Lus_13) treated with RNase R (DN+ RNR+) or DNase treatment alone (DN+). ss-: negative single stranded RNA, ss+: positive single stranded RNA, ds: double stranded RNA.

Supplementary Table S1. Mosquitoes analyzed in this study

year	month	place	No. mosquito	No. pool	RT-PCR (+) pools
2014	Apr	Siavonga	614	29	0
2014	Apr	Lusaka	111	8	1
2014	Jul	Lusaka	258	10	1
2014	Oct	Mongu	1463	66	3
2015	Feb	Lusaka	450	18	3
2015	Mar	Lusaka	1074	37	4
2015	June	Lusaka	72	4	1
2015	Apr	Livingstone	798	34	27
		Kazungla	91	19	3
2015	Nov	Chipata	307	17	0
2016	Mar	Siavonga	529	44	1
2016	May	Mongu	1037	67	5
2016	Nov	Kitwe	275	10	0
		Ndola	19	2	0
		Mwinilunga	49	13	2
2017	Apr	Livingstone	899	70	33
2017	May	Mongu	2406	138	11
2017	Nov	Isoka	267	47	10
		Mpulungu	383	36	3
2018	Aug	Mong	317	21	0
2018	Dec	Livingstone	590	27	24
		Sesheke	80	6	0
		Mongu	1434	78	2
2019	May	Mongu	1686	92	15
2022	Feb	Lusaka	627	18	0
2022	Mar	Lusaka	995	38	0
2022	Apr	Lusaka	450	15	1
		Total	17281	964	**150**

Supplementary Table S2.

Nucleotide BLAST analysis of nucleotide sequences of contigs from the RNA-seq sample (2015_Livingston_#15)

Query	Number of HSPs*	Lowest E-value	Accession (E-value)	Description (E-value)	Greatest identity %	Greatest HSP* length
L15RNR_S2_L001_R1_001_(paired)_contig_1	352	0	Y00055	Crithidia fasciculata ribosomal DNA repeat (complete)	99.65034965	1135
L15RNR_S2_L001_R1_001_(paired)_contig_2	33	0	JQ918037	Uncultured Orbus sp. clone GD5 16S ribosomal RNA gene, partial sequence	95.96273292	1288
L15RNR_S2_L001_R1_001_(paired)_contig_3	36	0	HQ154569	Providencia sp. R8-1A 16S ribosomal RNA gene, partial sequence	96.34235552	1366
L15RNR_S2_L001_R1_001_(paired)_contig_4	60	0	AM999887	Wolbachia endosymbiont of Culex quinquefasciatus Pel strain wPip complete genome	100	1424
L15RNR_S2_L001_R1_001_(paired)_contig_5	436	0	KU710348	Epichloe typhina strain E8 18S ribosomal RNA gene, internal transcribed spacer 1, 5.8S ribosomal RNA gene, internal transcribed spacer 2, and 28S ribosomal RNA gene, complete sequence	96.2962963	2554
L15RNR_S2_L001_R1_001_(paired)_contig_6	433	0	CP009056	Frischella perrara strain PEB0191, complete genome	96.22641509	1810
L15RNR_S2_L001_R1_001_(paired)_contig_7	235	0	CP017671	Providencia rettgeri strain RB151, complete genome	100	1939
L15RNR_S2_L001_R1_001_(paired)_contig_8	60	0	NR_074484	Rickettsia bellii strain RML369-C 16S ribosomal RNA gene, complete sequence	99.92205768	1288
L15RNR_S2_L001_R1_001_(paired)_contig_9	92	0	NR_076253	Wolbachia endosymbiont of Culex quinquefasciatus Pel strain wPip 23S ribosomal RNA, complete sequence	100	2703
L15RNR_S2_L001_R1_001_(paired)_contig_10	30	0	KT726939	Culex flavivirus isolate BR_SJRP_01_2012 polyprotein gene, complete cds	98.84649512	1127
L15RNR_S2_L001_R1_001_(paired)_contig_11	88	0	NR_076368	Rickettsia bellii strain RML369-C 23S ribosomal RNA gene, complete sequence	100	2683
L15RNR_S2_L001_R1_001_(paired)_contig_12	60	0	Y00055	Crithidia fasciculata ribosomal DNA repeat (complete)	100	1405
L15RNR_S2_L001_R1_001_(paired)_contig_13	30	0.00041	CP019609	Vagococcus penaei strain CD276, complete genome	94.28571429	81
L15RNR_S2_L001_R1_001_(paired)_contig_14	7	0.741352	CP009910	Borrelia chilensis strain VA1, complete genome	91.42857143	49
L15RNR_S2_L001_R1_001_(paired)_contig_15	30	0	JN793864	Uncultured Enterobacteriaceae bacterium clone Frankliniella schultzei10 16S ribosomal RNA gene, partial sequence	97.05438066	1324
L15RNR_S2_L001_R1_001_(paired)_contig_16	40	0	KP642114	Bunyaviridae environmental sample clone sraf.cpip_contig30 RNA-dependent RNA polymerase gene, complete cds	93.47826087	7265
L15RNR_S2_L001_R1_001_(paired)_contig_17	36	0	KX883008	Hubei mosquito virus 4 strain 3mos6213 hypothetical protein 1, hypothetical protein 2, and hypothetical protein 3 genes, complete cds	100	4900
L15RNR_S2_L001_R1_001_(paired)_contig_18	89	1.04E-86	GU188856	Culex quinquefasciatus mitochondrion, complete genome	100	185
L15RNR_S2_L001_R1_001_(paired)_contig_19	30	0	GQ165808	Culex flavivirus strain Uganda08 polyprotein gene, partial cds	98.34815756	2361
L15RNR_S2_L001_R1_001_(paired)_contig_20	30	0	KT726939	Culex flavivirus isolate BR_SJRP_01_2012 polyprotein gene, complete cds	98.54202401	1166
L15RNR_S2_L001_R1_001_(paired)_contig_21	30	0.014539	CP015199	Chryseobacterium sp. IHB B 10212 strain IHBB 10212, complete genome	94.11764706	77
L15RNR_S2_L001_R1_001_(paired)_contig_22	33	5.45E-49	KM817659	Wuhan Mosquito Virus 9 strain JX1-13 ORF1 (ORF1), ORF2 (ORF2), ORF3 (ORF3), glycoprotein (G), and RNA-dependent RNA polymerases genes, complete cds	93.93939394	894
L15RNR_S2_L001_R1_001_(paired)_contig_23	7	2.65E-21	KX882766	Hubei sobemo-like virus 41 strain 3mos6151 hypothetical protein genes, complete cds	91.17647059	721
L15RNR_S2_L001_R1_001_(paired)_contig_24	15	1.53E-12	KX883774	Hubei virga-like virus 23 strain mosHB236486 RdRp, hypothetical protein, putative glycoprotein, and hypothetical protein genes, complete cds	93.33333333	288
L15RNR_S2_L001_R1_001_(paired)_contig_25	10	0	KX883841	Wuhan Mosquito Virus 4 strain 3mos5038 segment PA polymerase PA gene, complete cds >gi|1110865693|gb|KX883866.1| Wuhan Mosquito Virus 4 strain XC3-4 segment PA polymerase PA gene, complete cds	100	1997
L15RNR_S2_L001_R1_001_(paired)_contig_26	32	0.383801	HG975440	Solanum pennellii chromosome ch01, complete genome	100	71
L15RNR_S2_L001_R1_001_(paired)_contig_27	3	0.109961	LK391419	Apteryx australis mantelli genome assembly AptMant0, scaffold scaffold27	93.33333333	41
L15RNR_S2_L001_R1_001_(paired)_contig_28	33	6.93E-36	KX883774	Hubei virga-like virus 23 strain mosHB236486 RdRp, hypothetical protein, putative glycoprotein, and hypothetical protein genes, complete cds	91.89189189	1161
L15RNR_S2_L001_R1_001_(paired)_contig_29	7	0.823189	LM257071	Strongylus vulgaris genome assembly S_vulgaris_Kentucky ,scaffold SVUK_scaffold0035204	93.5483871	45
L15RNR_S2_L001_R1_001_(paired)_contig_30	30	0.01936	LN713265	Cucumis melo genomic chromosome, chr_11	96.66666667	79
L15RNR_S2_L001_R1_001_(paired)_contig_31	40	0.001589	DQ265248	Plasmodium falciparum isolate Pf114_23 RIFIN (rif) gene, exon 2 and partial cds	100	61
L15RNR_S2_L001_R1_001_(paired)_contig_32	109	0	EU879060	Culex flavivirus strain CxFV-Mex07, complete genome	98.30508475	2711
L15RNR_S2_L001_R1_001_(paired)_contig_33	30	0	EU879060	Culex flavivirus strain CxFV-Mex07, complete genome	98.43847595	1600
L15RNR_S2_L001_R1_001_(paired)_contig_34	34	0	KM817623	Wuhan Mosquito Virus 4 strain XC3-4 PB1 (PB1) gene, complete cds	100	2400
L15RNR_S2_L001_R1_001_(paired)_contig_35	30	0.096274	LM716200	Protopolystoma xenopodis genome assembly P_xenopodis_South_Africa ,scaffold PXEA_contig0018497	96.66666667	61
L15RNR_S2_L001_R1_001_(paired)_contig_36	30	0.063979	AC246869	Solanum lycopersicum strain Heinz 1706 chromosome 2 clone hba-5f15 map 2, complete sequence	96.42857143	73
L15RNR_S2_L001_R1_001_(paired)_contig_37	8	0	KX883840	Wuhan Mosquito Virus 4 strain 3mos5038 segment PB2 polymerase PB2 gene, complete cds >gi|1110865691|gb|KX883865.1| Wuhan Mosquito Virus 4 strain XC3-4 segment PB2 polymerase PB2 gene, complete cds	98.34815756	2361
L15RNR_S2_L001_R1_001_(paired)_contig_38	5	9.49535	CP011906	Ovis canadensis canadensis isolate 43U chromosome 21 sequence	100	50
L15RNR_S2_L001_R1_001_(paired)_contig_39	30	0.01833	CP011906	Ovis canadensis canadensis isolate 43U chromosome 21 sequence	93.5483871	65
L15RNR_S2_L001_R1_001_(paired)_contig_40	52	8.8E-146	CP011374	Moraxella bovoculi strain 58069, complete genome	92.95774648	1346
L15RNR_S2_L001_R1_001_(paired)_contig_41	31	0.604553	HF933215	TPA_asm: Oryzias latipes strain Hd-rR, complete genome assembly, chromosome 9	100	56
L15RNR_S2_L001_R1_001_(paired)_contig_42	25	1.33E-15	KP642122	DsRNA virus environmental sample clone mill.culi_contig89 proline-alanine-rich protein gene, complete cds; and PArp-RdRp gene, partial cds	96.42857143	551
L15RNR_S2_L001_R1_001_(paired)_contig_43	43	0	KX353853	Pseudomonas fragi strain A22 plasmid pPFM, complete sequence	96.51741294	1483
L15RNR_S2_L001_R1_001_(paired)_contig_44	47	0	KX884635	Hubei reo-like virus 7 strain mosHB235771 RdRp gene, complete cds	100	3440
L15RNR_S2_L001_R1_001_(paired)_contig_45	30	0	EU879060	Culex flavivirus strain CxFV-Mex07, complete genome	97.53846154	1300
L15RNR_S2_L001_R1_001_(paired)_contig_46	31	0.0012037	XM_018190537	PREDICTED: Atta colombica uncharacterized LOC108685645 (LOC108685645), mRNA	96.55172414	96
L15RNR_S2_L001_R1_001_(paired)_contig_47	38	3.68E-10	KX883774	Hubei virga-like virus 23 strain mosHB236486 RdRp, hypothetical protein, putative glycoprotein, and hypothetical protein genes, complete cds	100	314
L15RNR_S2_L001_R1_001_(paired)_contig_48	15	0.178475	LN997849	Magnetospirillum sp. XM-1 genome assembly XM-1, plasmid : II	94.11764706	58
L15RNR_S2_L001_R1_001_(paired)_contig_49	33	1.02E-49	KX883774	Hubei virga-like virus 23 strain mosHB236486 RdRp, hypothetical protein, putative glycoprotein, and hypothetical protein genes, complete cds	96.66666667	1160
L15RNR_S2_L001_R1_001_(paired)_contig_50	26	3.57E-11	KX884812	Hubei bunya-like virus 5 strain SCM50476 RNA-dependent RNA polymerase gene, partial cds	96.42857143	144
L15RNR_S2_L001_R1_001_(paired)_contig_51	3	0.735786	KX882813	Hubei sobemo-like virus 21 strain CC64469 segment 2 hypothetical protein gene, partial cds	96.42857143	61
L15RNR_S2_L001_R1_001_(paired)_contig_52	31	0.017538	LT160000	Macaca fascicularis complete genome, chromosome chr1	100	66
L15RNR_S2_L001_R1_001_(paired)_contig_53	10	0.213655	LL188640	Heligmosomoides polygyrus genome assembly H_bakeri_Edinburgh ,scaffold HPBE_scaffold0000271	100	44
L15RNR_S2_L001_R1_001_(paired)_contig_54	30	0.061213	CP012201	Massilia sp. NR 4-1, complete genome	96.42857143	58
L15RNR_S2_L001_R1_001_(paired)_contig_55	33	0	KJ146684	Pseudomonas sp. LZ-E zonular occludens toxin gene, complete cds	96.8627451	765
L15RNR_S2_L001_R1_001_(paired)_contig_56	7	2.60285	CP006288	Saccharomyces cerevisiae YJM1311 chromosome III	93.33333333	44
L15RNR_S2_L001_R1_001_(paired)_contig_57	32	0	CP005092	Sphingobium sp. TKS plasmid pTK8, complete sequence	95.42097489	699

* HSP : High-scoring Segment Pairs for the query sequence

Supplementary Table S3.

Viral proteins BLASTx analysis of contigs from the RNA-seq sample (2015_Livingston_#15)

Query	Number of HSPs*	Lowest E-value	Accession (E-value)	Description (E-value)	Greatest identity %	Greatest HSP* length
L15RNR_S2_L001_R1_001_(paired)_contig_1	0		not available	not available		
L15RNR_S2_L001_R1_001_(paired)_contig_2	0		not available	not available		
L15RNR_S2_L001_R1_001_(paired)_contig_3	7	8.11E-05	AIM51786	hypothetical protein HQ81_0265 [Dickeya phage phiDP23.1]	100	111
L15RNR_S2_L001_R1_001_(paired)_contig_4	1	4.26E-11	AIM51786	hypothetical protein HQ81_0265 [Dickeya phage phiDP23.1]	55.3571429	56
L15RNR_S2_L001_R1_001_(paired)_contig_5	1	0.016698	QDH88209	hypothetical protein H2RhizoLitter494174_000002, partial [Mitovirus sp.]	72.4137931	29
L15RNR_S2_L001_R1_001_(paired)_contig_6	1	3.31E-11	AIM51792	hypothetical protein HQ81_0272 [Dickeya phage phiDP23.1]	64.5833333	48
L15RNR_S2_L001_R1_001_(paired)_contig_7	0		not available	not available		
L15RNR_S2_L001_R1_001_(paired)_contig_8	1	1.86907	AZF88202	minor tail protein [Rothia phage Spartoi]	29.6296296	54
L15RNR_S2_L001_R1_001_(paired)_contig_9	0		not available	not available		
L15RNR_S2_L001_R1_001_(paired)_contig_10	30	0	AML84517	polyprotein [Culex flavivirus]	99.2	375
L15RNR_S2_L001_R1_001_(paired)_contig_11	0		not available	not available		
L15RNR_S2_L001_R1_001_(paired)_contig_12	4	3.28785	YP_007675561	terminase small subunit [Synechococcus phage S-RIM2 R1_1999] >gb|AGH06781.1|	35.483871	60
L15RNR_S2_L001_R1_001_(paired)_contig_13	17	8.93E-21	YP_009104381	nonstructural protein [Fako virus] >gb|AIW39873.1|	43.3333333	589
L15RNR_S2_L001_R1_001_(paired)_contig_14	3	4.82E-66	API61885	hypothetical protein, partial [Salarivirus Mos8CM0]	28.7313433	529
L15RNR_S2_L001_R1_001_(paired)_contig_15	1	9.097	VFR13000	hypothetical protein SPFM12_00003 [Salmonella phage SPFM12]	48.2758621	29
L15RNR_S2_L001_R1_001_(paired)_contig_16	32	0	AJT39594	RNA-dependent RNA polymerase [Bunyaviridae environmental sample]	97.2416632	2429
L15RNR_S2_L001_R1_001_(paired)_contig_17	31	0	APG76308	hypothetical protein 1 [Hubei mosquito virus 4]	99.5798319	1006
L15RNR_S2_L001_R1_001_(paired)_contig_18	0		not available	not available		
L15RNR_S2_L001_R1_001_(paired)_contig_19	30	0	ACV04604	polyprotein, partial [Culex flavivirus]	99.6183206	786
L15RNR_S2_L001_R1_001_(paired)_contig_20	30	0	ACJ64914	polyprotein [Culex flavivirus]	99.5037221	403
L15RNR_S2_L001_R1_001_(paired)_contig_21	13	3.94E-88	AIW39886	non-structural protein [Fako virus]	32.5581395	1163
L15RNR_S2_L001_R1_001_(paired)_contig_22	41	0	QEM39120	RNA-dependent RNA polymerase [Guadeloupe Culex rhabdovirus]	99.7193639	2138
L15RNR_S2_L001_R1_001_(paired)_contig_23	30	0	ASA47391	hypothetical protein [Culex luteo-like virus] >gb|ASA47482.1| hypothetical protein [Culex luteo-like virus]	77.7777778	477
L15RNR_S2_L001_R1_001_(paired)_contig_24	30	0	QGA70941	hypothetical protein [Rinkaby virus]	75.3424658	949
L15RNR_S2_L001_R1_001_(paired)_contig_25	38	0	APG77862	polymerase PA [Wuhan Mosquito Virus 4] >gb|APG77888.1| polymerase PA [Wuhan Mosquito Virus 4]	100	724
L15RNR_S2_L001_R1_001_(paired)_contig_26	4	1.5E-13	YP_009104384	clamp protein [Fako virus] >pdb|6DJY|A Chain A,	40	281
L15RNR_S2_L001_R1_001_(paired)_contig_27	30	2.78E-89	AUF41956	RNA-dependent RNA polymerase [Phytomonas sp. TCC231 leishbunyavirus 1]	37.5722543	742
L15RNR_S2_L001_R1_001_(paired)_contig_28	57	0	QGA70943	hypothetical protein [Rinkaby virus]	87.6971609	1197
L15RNR_S2_L001_R1_001_(paired)_contig_29	0		not available	not available		
L15RNR_S2_L001_R1_001_(paired)_contig_30	26	0	QEM39081	VP1 [Guadeloupe Culex rhabdovirus] >gb|QEM39091.1|	100	513
L15RNR_S2_L001_R1_001_(paired)_contig_31	33	7.4E-164	YP_009104386	major capsid protein [Fako virus] >pdb|6DJY|B Chain B,	31.2977099	1187
L15RNR_S2_L001_R1_001_(paired)_contig_32	30	0	ASL70040	polyprotein [Culex flavivirus]	100	688
L15RNR_S2_L001_R1_001_(paired)_contig_33	30	0	ACJ64914	polyprotein [Culex flavivirus]	100	546
L15RNR_S2_L001_R1_001_(paired)_contig_34	30	0	AJG39092	PB1 [Wuhan Mosquito Virus 4]	99.8726115	788
L15RNR_S2_L001_R1_001_(paired)_contig_35	34	5.5E-175	YP_443939	VP5 [Aedes pseudoscutellaris reovirus] >sp|Q2Y0E6.1|	35.2112676	961
L15RNR_S2_L001_R1_001_(paired)_contig_36	31	0	AXQ04767	hypothetical protein [Culex Bunya-like virus]	94.2028986	414
L15RNR_S2_L001_R1_001_(paired)_contig_37	30	0	APG77861	polymerase PB2 [Wuhan Mosquito Virus 4] >gb|APG77887.1| polymerase PB2 [Wuhan Mosquito Virus 4]	99.6168582	791
L15RNR_S2_L001_R1_001_(paired)_contig_38	3	1.39E-37	ASA47332	glycoprotein [Wuhan Mosquito Virus 6]	29.2682927	321
L15RNR_S2_L001_R1_001_(paired)_contig_39	20	1.14E-44	YP_443940	VP6 [Aedes pseudoscutellaris reovirus] >sp|Q2Y0E5.1|	32.2033898	436
L15RNR_S2_L001_R1_001_(paired)_contig_40	12	6.82E-59	AXN57518	MULTISPECIES	57.7464789	449
L15RNR_S2_L001_R1_001_(paired)_contig_41	46	5.27E-11	APG79301	RNA-dependent RNA polymerase, partial [Hubei bunya-like virus 5]	54.2857143	277
L15RNR_S2_L001_R1_001_(paired)_contig_42	30	0	AJT39583	PArp-RdRp, partial [dsRNA virus environmental sample]	70.8737864	690
L15RNR_S2_L001_R1_001_(paired)_contig_43	11	0.322581	QBI77694	hypothetical protein [Pseudomonas aeruginosa] >gb|QBI77694.1|	38.9830508	71
L15RNR_S2_L001_R1_001_(paired)_contig_44	44	0	AZL88835	RNA-dependent RNA polymerase [Hubei reo-like virus 7]	100	1169
L15RNR_S2_L001_R1_001_(paired)_contig_45	30	0	ACJ64914	polyprotein [Culex flavivirus]	98.0997625	459
L15RNR_S2_L001_R1_001_(paired)_contig_46	3	1.95E-12	AIW39854	non-structural protein, partial [Fako virus] >gb|AIW39855.1|	27.9761905	241
L15RNR_S2_L001_R1_001_(paired)_contig_47	51	0	QGA70941	hypothetical protein [Rinkaby virus]	85.1905451	2069
L15RNR_S2_L001_R1_001_(paired)_contig_48	1	1.93E-21	APG77120	hypothetical protein, partial [Hubei narna-like virus 16]	31.4079422	257
L15RNR_S2_L001_R1_001_(paired)_contig_49	34	0	QGA70943	hypothetical protein [Rinkaby virus]	94.3722944	1200
L15RNR_S2_L001_R1_001_(paired)_contig_50	30	0	AUF41956	RNA-dependent RNA polymerase [Phytomonas sp. TCC231 leishbunyavirus 1]	43.9655172	1046
L15RNR_S2_L001_R1_001_(paired)_contig_51	30	5.9E-63	ASA47393	capsid protein [Culex luteo-like virus] >gb|ASA47484.1| capsid protein [Culex luteo-like virus]	55.1546392	194
L15RNR_S2_L001_R1_001_(paired)_contig_52	3	1.81E-12	AUF41956	RNA-dependent RNA polymerase [Phytomonas sp. TCC231 leishbunyavirus 1]	26.7558528	273
L15RNR_S2_L001_R1_001_(paired)_contig_53	14	7.68E-95	QGA70933	hypothetical protein [Osta virus]	47.6608187	357
L15RNR_S2_L001_R1_001_(paired)_contig_54	0		not available	not available		
L15RNR_S2_L001_R1_001_(paired)_contig_55	23	2.11E-06	AWY09612	hypothetical protein [Xanthomonas campestris] >gb|AAY49173.1| pl	32.9411765	149
L15RNR_S2_L001_R1_001_(paired)_contig_56	30	0	ASA47413	RdRp [Culex mononega-like virus 2]	71.6981132	374
L15RNR_S2_L001_R1_001_(paired)_contig_57	2	9.79E-12	YP_001449258	transcriptional regulator	34.5454545	110

* HSP : High-scoring Segment Pairs for the query sequence

Supplementary Table S4. Accession no. of bunyavirus sequences used in this study

Viruses identified in this study	segment	isolate	Accession no.	BioSample
Culex tenui-like virus	L	zmq19mong-50	LC772127	SAMD00624236
Culex tenui-like virus	M	zmq19mong-50	LC772128	SAMD00624236
Culex tenui-like virus	3	zmq19mong-50	LC772129	SAMD00624236
Culex tenui-like virus	4	zmq19mong-50	LC772130	SAMD00624236
Culex hudovirus	L	zmq18mong-50	LC772131	SAMD00624237
Culex hudovirus	M	zmq18mong-50	LC772132	SAMD00624237
Culex hudovirus	S	zmq18mong-50	LC772133	SAMD00624237
Culex bunyavirus 2	L	zmq15Liv-15	LC772134	SAMD00624238
Culex bunyavirus 2	M	zmq15Liv-15	LC772135	SAMD00624238
Culex bunyavirus 2	S	zmq15Liv-15	LC772136	SAMD00624238
Anopheles phasivirus 1	L	zmq16siav-40	LC772137	SAMD00624239
Anopheles phasivirus 1	M	zmq16siav-40	LC772138	SAMD00624239
Anopheles phasivirus 1	S	zmq16siav-40	LC772139	SAMD00624239
Anopheles phasivirus 2	L	zmq15Liv-29	LC772140	SAMD00624240
Anopheles phasivirus 2	M	zmq15Liv-29	LC772141	SAMD00624240
Anopheles phasivirus 2	S	zmq15Liv-29	LC772142	SAMD00624240
Anopheles bunyavirus 1	L	zmq15Liv-29b	LC772143	SAMD00625169
Anopheles bunyavirus 1	M	zmq15Liv-29b	LC772144	SAMD00625169
Anopheles bunyavirus 1	S	zmq15Liv-29b	LC772145	SAMD00625169
Culex goukovirus 1	L	zmq16mwi-21	LC772146	SAMD00624241
Culex goukovirus 1	M	zmq16mwi-21	LC772147	SAMD00624241
Culex goukovirus 1	S	zmq16mwi-21	LC772148	SAMD00624241
Coquillettidia bunyavirus	L	zmq19mong-40	LC772149	SAMD00624242
Coquillettidia bunyavirus	M	zmq19mong-40	LC772150	SAMD00624242
Coquillettidia bunyavirus	S	zmq19mong-40	LC772151	SAMD00624242
Culex pheuivirus 3	S	zmq18mong-50d	LC772152	SAMD00625167
Culex leishbunyavirus 1	L	zmq15Liv-15c	LC772153	SAMD00625171
Culex leishbunyavirus 2	L	zmq15Liv-15b	LC772154	SAMD00625168
Culex leishbunyavirus 3	L	zmq18mong-50b	LC772155	SAMD00625165
Kristianstad virus	L	zmq18mong-50c	LC772156	SAMD00625166
Herbevirus herberti	L	zmq16mwi-21b	LC772157	SAMD00625170
Herbevirus herberti	M	zmq16mwi-21b	LC772158	SAMD00625170
Herbevirus herberti	S	zmq16mwi-21b	LC772159	SAMD00625170
Culex phenuivirus 1	L	zmq17mong-17	LC772907	SAMD00628353
Culex phenuivirus 1	S	zmq17mong-17	LC772908	SAMD00628353
Aedes phasivirus	L	zmq17mpu-71	LC772909	SAMD00623747
Culex goukovirus 1	L	zmq16mwi-21	LC772910	SAMD00623748
Culex goukovirus 2	L	zmq14mong-58	LC772911	SAMD00623749
Culex goukovirus 3	L	zmq19mong-51	LC772912	SAMD00623750
Mansonia goukovirus	L	zmq19mong-35	LC772913	SAMD00623751
Culex phenuivirus 2	L	zmq17mong-117	LC772914	SAMD00623752
Coquillettidia phenuivirus	L	zmq19mong-65	LC772915	SAMD00623753
Coquillettidia phenuivirus	L	zmq17iso-43	LC772916	SAMD00623754
Anopheles bunyavirus 1	L	zmq19mong-75	LC772917	SAMD00623755
Anopheles bunyavirus 1	L	zmq17Liv-46	LC772918	SAMD00623756
Mansonia bunyavirus	L	zmq19mong-34	LC772919	SAMD00623757
Coquillettidia bunyavirus	L	zmq17iso-22	LC772920	SAMD00623758
Anopheles bunyavirus 2	L	zmq17Liv-51	LC772921	SAMD00623759
Aedes bunyavirus	L	zmq17Liv-4	LC772922	SAMD00623760
Culex bunyavirus 2	L	zmq14mong-45	LC772923	SAMD00623761
Culex bunyavirus 2	L	zmq17Liv-1	LC772924	SAMD00623762
Culex bunyavirus 2	L	zmq15Lus6-3	LC772925	SAMD00623763

L segment RdRP	Accession no.
Rift Valley fever virus	DQ375402
Toscana virus	X68414
Sandfly Sicilian Turkey virus	NC_015412
Blacklegged tick phlebovirus 1	KX184200
Dabieshan Tick Virus	KM817666
Uukuniemi virus	KM114249
Severe fever with thrombo-cytopenia syndrome virus	KF887445
Kuriyama virus	LC133178
Bhanja virus	NC_027140
American dog tick phlebovirus	KM048311
Kaisodi virus	NC_040494
Kabuto mountain virus	NC_036604
Wenling crustacean virus 7	KU976394
Hubei lepidoptera virus 1	KX884772
Pink bollworm virus 3	MN164622
Hubei diptera virus 5	NC_032276
Whenzhou Shrimp Virus 1	NC_031287
Phasi Charoen-like virus	KM001085
Wutai Mosquito virus	KM817700
Badu virus isolate	KT693187
Wuhan horsefly Virus	NC_031313
Fitzroy Crossing tenui-like virus 1	QLJ83469
Ramu stunt virus	KR094115
Melon chlorotic spot virus	NC_040450
Rice grassy stunt virus	NC_002323
Rice stripe virus	JQ927433
Wuhan Insect virus 1	KM817691
Shahe heteroptera virus 3	KX924627
Yichang Insect virus	KM817703
Cumuto virus	NC_043045
Gouleako virus	NC_043051
Xinzhou bunya-likevirus 1	KX884868
Watermelon crinkle leaf-ass virus1	KY781184
Shuangao Insect Virus 3	KM817681
Zhee Mosquito virus	KM817705
Salarivirus	KX924627
Xinzhou Mosquito Virus	KM817701
Culex Bunya-like virus	MH188002
Culex pseudovishnui bunya-like virus	LC514293
Bunyaviridae environmental sample	KP642114
Culex Bunyavirus 2	MH188052
Wuhan Spider Virus	KM817699
Leptomonas moramango leishbunyavirus isolate LepmorLBV1a-L	KX280012
Leptomonas moramango leishbunyavirus isolate LepmorLBV1b-L	KX280015
Phytomonas sp. leishbunyavirus 1	KY322667
Crithidia ZM virus	KX373293
Kristianstad virus	MK440644
Crimean-Congo hemorrhagic fever virus	AY422209
Herbert virus	JQ659256
La Crosse virus	GU206125

M segment GP	Accession no.
Rift Valley fever virus	DQ380198
Toscana virus	KX010933
Sandfly Sicilian Turkey virus	NC_015411
Uukuniemi virus	KM114250
Severe fever with thrombo-cytopenia syndrome virus	KF887440
Kuriyama virus	LC133179
Bhanja virus	NC_027141
Kaisodi virus	NC_040493
Mourilyan virus	MT241518
Whenzhou Shrimp Virus 1	NC_031292
Hubei lepidoptera virus 1	NC_032257
Hubei diptera virus 5	NC_032277
Pink bollworm virus 3	MN164621
Shahe heteroptera virus 3	NC_032142
Phasi Charoen-like virus	KM001086
Badu virus	KT693188
Yichang Insect virus	KM817730
Cumuto virus	KF543245
Gouleako virus	HQ541737
Shuangao Insect Virus 3	KM817716
Salarivirus	KX924628
Narangue virus	MN661014
Culex pseudovishnui bunya-like virus	LC514292
Culex phasma-like virus	MF176314
Wuhan horsefly Virus	NC_031316
Fitzroy Crossing tenui-like virus 1	MT498813
Herbert virus strain	KF590579

S segment NP	Accession no.
Rift Valley fever virus	DQ380171
Toscana virus	KX010932
Sandfly Sicilian Turkey virus	NC_015413
Candiru virus	NC_015375
Kuriyama virus	LC133180
Kaisodi virus	NC_040492
Uukuniemi virus	KM114251
Kabuto mountain virus	LC153713
Severe fever with thrombo-cytopenia syndrome virus	AB985578
American dog tick phlebovirus	KM048312
Bole Tick Virus 1	KM817731
Lihan Tick Virus	KM817736
Bhanja virus	NC_027142
Blacklegged tick phlebovirus 1	KX184201
Yichang Insect virus	KM817763
Shahe heteroptera virus 3	NC_032141
Cumuto virus	KF543246
Cumuto virus	KF543245
Gouleako virus	HQ541736
Whenzhou Shrimp Virus 1	NC_031290
Phasi Charoen-like virus	KM001087
Badu virus	KT693189
Laurel Lake virus	NC_043681
Hubei bunya-like virus 13	KX884785
Hubei bunya-like virus 3	KX884779
Pink bollworm virus 3	MN164620
Shuangao Insect Virus 3	KM817742
Hubei lepidoptera virus 1	KX884774
Narangue virus	MN661014
Salarivirus	KX924629
Culex pseudovishnui bunya-like virus	LC516823
Rice stripe virus	JP927420
Wuhan horsefly Virus	KM87751
Ramu stunt virus	KR094118
Rice grassy stunt virus	KF438679
Fitzroy Crossing tenui-like virus 1	MT498815
La Crosse virus	GU206123
Herbert virus strain	KF590578
